# Recent Advances in *Moringa* Multi-Omics Research: Driving Breeding Innovation and Application Prospects

**DOI:** 10.3390/biology15131040

**Published:** 2026-06-30

**Authors:** Yanni Liu, Leng Wang, Mingxia Xiao, Jiming Long, Haiquan Li, Baolan Ren, Zubing Zhang

**Affiliations:** Yunnan Institute of Tropical Crops, Jinghong 666100, China; liuyn9523@163.com (Y.L.); 15894304650@163.com (L.W.); mmswxxa8346@163.com (M.X.); ljm110112@163.com (J.L.); 13578150901@139.com (H.L.)

**Keywords:** functional genomics, molecular breeding, *Moringa*, systems biology, sustainable agriculture

## Abstract

*Moringa*, a versatile tree species, has seen an increasing diversification of breeding objectives due to its rich nutritional value and potential applications in ecological restoration. The emergence and integration of multi-omics technologies have provided a revolutionary systems-level research framework for elucidating the fundamental biological mechanisms underlying *Moringa*’*s* agronomic traits and nutritional characteristics. This review systematically analyzes the application prospects and omics significance of *Moringa* based on research trends, and explores in depth the progress made in various omics studies of *Moringa*: the fourth iteration of the genome has identified specific genes encoding heat shock proteins (HSPs) in *Moringa*; phenomics reveals differential expression of *Moringa* under different environmental conditions; and the transcriptomics and metabolomics elucidate differential regulatory networks across different tissues and environments. In the future, multi-omics technologies can be integrated: genomics can further identify rare alleles and localize genetic loci for key agronomic traits; transcriptomics combined with epigenomics can elucidate the spatial regulation of gene expression and epigenetic mechanisms; and proteomics and metabolomics can be integrated to validate pathways and provide targets for improvement. Throughout the process, a high-throughput phenotyping platform utilizing drones will be introduced to dynamically monitor agronomic traits, enabling efficient breeding and accelerating genetic improvement.

## Simple Summary

*Moringa*, a highly versatile tree, has leaves rich in protein and vitamins, and seeds capable of purifying wastewater and improving poor soil; it holds immense potential for addressing global malnutrition and combating climate change. However, the breeding of new varieties currently relies primarily on traditional methods, which are slow and inefficient, making it difficult to meet diverse demands. This paper systematically reviews global research progress on *Moringa* over the past 20 years, analyzes growth variations across different varieties and environments, and examines findings regarding associated genes and components, thereby explaining the reasons behind *Moringa*’*s* high nutritional value and strong stress tolerance. It concludes that the integration of multiple research approaches, alongside the introduction of emerging technologies, is still required to develop *Moringa* varieties suitable for diverse applications such as food, feed and ecological restoration.

## 1. Introduction

**Figure 1:**
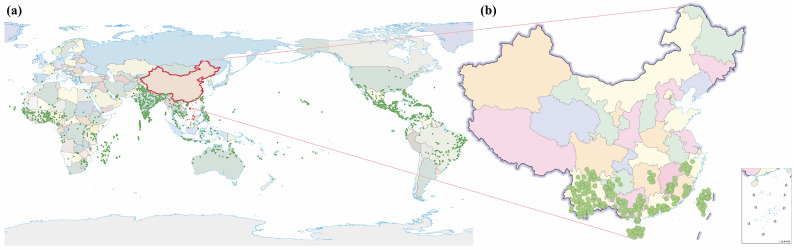
Global distribution of *Moringa*. (**a**) Worldwide distribution of *Moringa* (GBIF Occurrence Download. Available online: https://doi.org/10.15468/dL.3arecp (accessed on 17 November 2025) [6], (**b**) Distribution of *Moringa* in China (scale 1:7,400,000). The distribution map is based on GBIF occurrence records, occurrence data should therefore be interpreted with sampling bias in mind.

*Moringa* is a deciduous tree belonging to the monotypic family Moringaceae, with approximately 13 species distributed worldwide [1]. Native to the arid and semi-arid regions of the Indian subcontinent and Africa, *Moringa oleifera* has become extensively naturalized through cultivation and is now primarily found in the tropical and subtropical areas of Asia and Africa [2,3,4,5]. Initially introduced to China in Taiwan, its cultivation range has subsequently expanded to numerous southern provinces (Figure 1).

*Moringa* is a versatile tree species with utility across all its tissues. Young shoots, characterized by rapid growth, low fiber content, and tenderness, constitute the primary edible portion of the plant [7]. Leaves are rich in proteins, minerals, vitamins, and bioactive compounds, such as polyphenols and polysaccharides, which facilitate their extensive utilization in pharmaceutical, food [8], and feed additive industries [9]. Roots accumulate bioactive alkaloids [10], while oil extracted from seeds contains a high concentration of oleic acid (>70%) [11], and possesses excellent oxidative stability and multifunctional properties [12], including the promotion of wound healing [13]. From an ecological perspective, *Moringa* improves soil structure and enhances water retention [14], thereby increasing its value in agroforestry systems.

With the widespread recognition of *Moringa*’*s* multifaceted value, there is a growing demand in the market for varieties with high levels of bioactive compounds and strong stress tolerance. However, current breeding methods rely primarily on traditional hybrid breeding and phenotypic screening, which struggle to meet diverse market demands; thus, there is an urgent need to establish an efficient modern breeding system. The integration of multi-omics research provides precise technical support to meet the targeted requirements of *Moringa* breeding. This paper comprehensively reviews the current application areas of *Moringa* and the latest advances in multi-omics research, providing a theoretical foundation for its diverse applications, and further outlines future research directions. It aims to lay a scientific foundation for various breeding approaches in *Moringa*, while providing technical support for germplasm resource innovation and the formulation of diversified industrial strategies.

## 2. Current Trends in *Moringa* Research

To elucidate the research trajectory and prevailing trends in the field of *Moringa*, a bibliometric analysis was conducted on the relevant literature. Bibliographic records were retrieved from the Web of Science (WoS) Core Collection using the topic query TS = (“*Moringa*”) and were restricted to research articles and reviews published between 2005 and 2025; the search was last updated in January 2026, and only English-language records were retained after the removal of duplicates. The retrieved records were imported into CiteSpace, and country co-occurrence, keyword clustering, keyword timeline, and citation-burst analyses were performed to obtain a comprehensive overview of the literature on *Moringa* [15,16,17].

**Figure 2:**
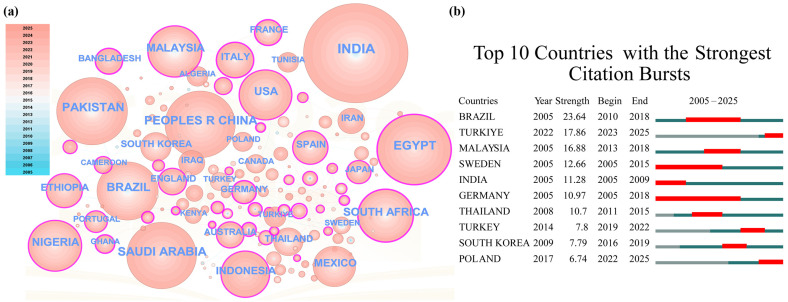
Country clustering map of *Moringa* publications in the Web of Science database. (**a**) Country clustering map of research articles and reviews published from 2005 to 2025 (N = 152, E = 231, density = 0.0201). Node size indicates frequency of occurrence, and edge thickness represents centrality levels. (**b**) Visualization of burst trends by country; strength > 3.0 indicates a high burst intensity.

India, the country of origin of *Moringa* [1,2], emerged as the sole nation with an output exceeding 1000 publications, totaling 1363 articles. Egypt (673 articles), Pakistan (658 articles), Saudi Arabia (637 articles), China (633 articles), and Brazil (516 articles) contributed over 500 publications each (Figure 2a). Citation burst analysis identified Brazil as the most prominent contributor, with a burst intensity of 23.64. Turkey (2023–2025) and Poland (2022–2025) are currently experiencing active and sustained research surges, with both nations demonstrating burst intensities exceeding 6. This emerging geographical distribution of research highlights the significant global increase in scientific interest in *Moringa* worldwide. This growing interest can be attributed to *Moringa*’*s* highly bioactive compounds and its resistance to abiotic stresses, further underscoring the need to employ advanced genomics tools to decipher the complex genetic architecture underlying *Moringa*’*s* key traits.

**Figure 3:**
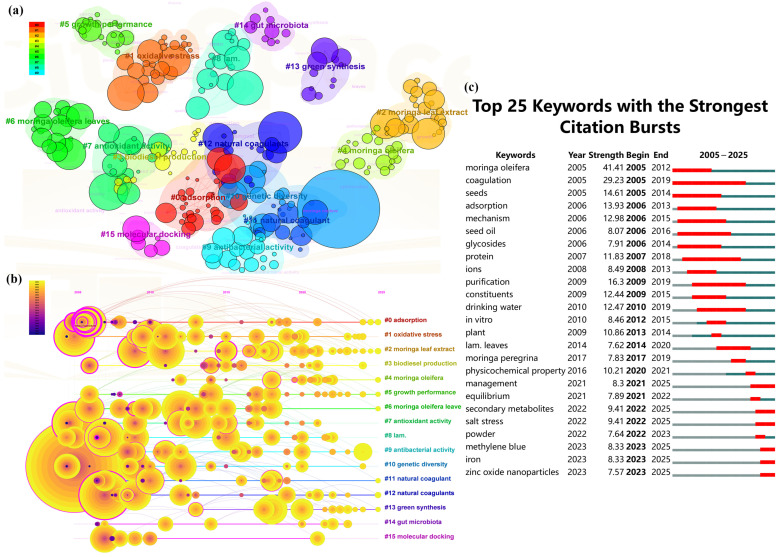
Keyword clustering network map of *Moringa* literature based on Web of Science (WoS) journals. (**a**) Keyword clustering diagram of research articles and reviews published from 2020 to 2025 (N = 288, E = 348, density = 0.0084); (**b**) Keyword timeline clustering network diagram; (**c**) Visualization analysis of keyword burst trends, where strength > 3.0 indicates high burst intensity.

Keyword clustering analysis effectively illuminated the distribution and research hotspots of *Moringa* studies, providing a clear basis for understanding the field’s focal points. Research on *Moringa* has progressed through distinct phases. Initial investigations concentrated on the plant itself; this was followed by a shift toward efficacy-driven studies, and more recently, a growing emphasis on industrial applications. The application of keyword timeline clustering reveals fluctuations in research emphasis across different periods (Figure 3a,b). Early work was predominantly concerned with fundamental aspects such as “genetic diversity.” Subsequently, the focus deepened to explore the underlying mechanisms and functionalities, incorporating approaches such as “molecular docking” and examining processes related to “oxidative stress” and “antibacterial activity.” As knowledge of *Moringa*’*s* functional properties has expanded, its potential applications have diversified, and current research trends prominently feature keywords such as “moringa leaf extract,” “natural coagulants,” and “gut microbiota,” reflecting this translation toward practical utilization.

A comprehensive analysis revealed that “coagulation” was the most enduring and prominent research theme, exhibiting a citation burst intensity of 29.23 from 2005 to 2019. This sustained attention underscores the current prominence of *Moringa* as a prime bio-based alternative for water treatment applications. While early investigations were confined to moringa seeds, recent research has broadened the scope to encompass all parts of the plant (Figure 3c). Analysis of current high-frequency emerging keywords highlights several research convergence points, including “salt stress,” “secondary metabolites,” and “nanoparticles” (e.g., zinc oxide). Research into *Moringa* omics is gradually gaining momentum. By integrating genomic data with transcriptomic profiles under stress conditions, key regulatory genes and metabolic pathways involved in drought and salt tolerance can be identified and modulated. For example, genetic diversity analyses using DArTSeq SNP markers have revealed significant heterozygosity within *Moringa* populations. This can be further explored through the mapping of expression quantitative trait loci (eQTLs), thereby establishing links between genetic variation and the expression of stress-response genes [18]. These are critical for addressing global challenges related to climate resilience and advanced materials science, further emphasizing the high adaptability of *Moringa* across multiple utilization scenarios.

To fully harness the potential of *Moringa*, a transition from fragmented descriptive studies to comprehensive systems-level analyses coupled with targeted breeding for application scenarios is imperative. In this context, multi-omics technologies are crucial for elucidating the underlying molecular mechanisms; they provide a valuable molecular framework for analyzing the specific pathways through which *Moringa* exerts its ecological restoration functions and for optimizing key metabolic synthesis pathways. This will drive the *Moringa* industry forward from traditional breeding toward future molecular breeding and customized applications.

## 3. Progress in Multi-Omics Research on *Moringa*

### 3.1. Genomics Reveals Genetic Diversity

DNA molecular markers are crucial for identifying *Moringa* germplasm resources, breeding varieties, and managing cultivation. Researchers have employed various techniques to analyze the genetic diversity and structure of *Moringa*, including Random Amplified Polymorphic DNA (RAPD) [19], inter-simple sequence repeat (ISSR) [20], sequence-characterized amplified region (SCAR) [21], Simple Sequence Repeat (SSR) [22], Single-Nucleotide Polymorphism (SNP) [18], and start codon-targeted polymorphism (ScoT) [23]. These studies have elucidated the association between genetic variation and geographic origin, providing a scientific foundation for the conservation, classification, and rational utilization of genetic resources [2]. Furthermore, they aid in identifying superior genotypes, offer references for subsequent targeted introduction and cultivation plans, and establish a critical genetic knowledge base for future molecular breeding endeavors and the sustainable development of the *Moringa* industry.

Genomic libraries form a vital foundation connecting genotype with phenotype, thereby accelerating the development of new *Moringa* germplasm resources and their industrial applications. Tian et al. [24] first deciphered the genome of *Moringa oleifera*; they reported its genome size (2*n* = 28) as 315.6 Mb and suggested that the plant’s rapid growth, high seed yield, and strong drought tolerance might be associated with its relatively small genome. Between 2019 and 2022, three genomic assembly efforts for *Moringa oleifera* were undertaken [25,26,27], which achieved significant improvements including genome size, contiguity, and completeness of protein-coding gene annotation in key assembly metrics (Table 1). These iterative refinements have yielded genomic resources of increasing quality and utility. The resultant contiguous and well-annotated reference genomes have facilitated the subsequent development of improved varieties with enhanced yields and stress tolerance. Moreover, they provide an indispensable foundation for elucidating the regulatory networks governing biosynthesis within the plant, including genome-wide selection and future CRISPR/Cas-based gene editing techniques.

From 2015 to 2022, the sequencing coverage and read length of *Moringa* genome sequencing technology continued to improve, and the quality of the assembly gradually improved as a result; however, as of 2022, 7.3% of the sequence remained unanchored to chromosomes, and inconsistencies in gene annotation were particularly pronounced. The number of protein-coding genes differed by nearly a factor of two, and significant variation was also observed in the average number of exons, the proportion of repetitive sequences, and the number of non-coding RNAs. Comparative genomic analyses revealed that gene family expansions and contractions in *Moringa* reflect adaptive evolution in response to environmental pressures. Tian et al. [24] identified a suite of four *SKP1* genes and 18 F-box domain-encoding genes, potentially unique to *Moringa*. Furthermore, copy number variations (CNVs) in the *BAK1* gene and genes encoding heat shock proteins (HSPs) have been observed in *Moringa* plants [27]. These specific genomic alterations underpin key agronomic traits, including rapid growth and enhanced tolerance to drought and heat stress. Studies have indicated that expanded gene families in the *Moringa* genome are predominantly associated with chloroplast and photosynthetic functions [28], whereas contracted families include cytochrome P450 enzymes and *SOT1* [27]. Further investigation has demonstrated that *MoTPS1* exhibits TPS activity, suggesting a potential role for this gene family in sugar metabolism [29].

However, the *Moringa* genome still has many shortcomings. For example, additional genome completeness assessment metrics, such as the Benchmarking Universal Single-Copy Orthologs (BUSCO) score, are needed to ensure genome coverage. The high heterozygosity of the *Moringa* genome also makes it prone to assembly errors and the omission of haplotype sequences. Furthermore, regarding key traits in moringa leaves—such as micronutrient content, accumulation of secondary metabolites, and stress tolerance—current research on the identification of regulatory genes [30] remains limited. Similarly, miRNA target genes have only undergone preliminary identification and analysis [31], necessitating further in-depth investigation in these areas. It is crucial not only to elucidate the genetic basis of these polygenic quantitative traits but also to conduct Structural Variant (SV) analysis. In particular, the construction of a *Moringa* pan-genome from diverse accessions would capture the core and dispensable gene sets and systematically resolve large structural variants, including presence/absence variation (PAV) and copy-number variation (CNV), that are frequently associated with stress-tolerance and nutritional traits but are missed by single-reference, SNP-based approaches [32]. Comparative genomics studies among different species within the *Moringa* genus are also necessary to better elucidate genetic diversity during the evolution of *Moringa*.

Future applications of emerging genomic technologies will enable a more profound understanding of the *Moringa* genome. Genome-wide association studies (GWASs) hold promise for the precise localization of genetic loci controlling complex agronomic and nutritional traits. Advances in single-cell sequencing technology can reveal the regulatory mechanisms underlying these phenotypes. CRISPR/Cas9 gene editing technology reduces the costs of large-scale breeding and phenotypic analysis, thereby significantly enhancing the efficiency of *Moringa* genetic improvement efforts. This is crucial for the subsequent development of superior *Moringa* varieties and the expansion of their industrial applications. It should be noted, however, that the application of CRISPR/Cas9 in *Moringa* remains at a prospective stage, as no efficient and stable genetic transformation or in vitro regeneration system has yet been established for this species, which currently constitutes the principal bottleneck for genome editing [33].

### 3.2. Phenomics Reveals Trait Development

Genetic, environmental, and signal transduction differences can be reflected in the phenotypes. Appropriate phenotypic data allow precise localization of specific genomic and spatial environmental factors [34]. Integrating phenomics with other omics approaches can advance the sustainable utilization and breeding optimization of *Moringa*.

Karunakar et al. [35] demonstrated that the key agronomic traits for yield evaluation and breeding selection include single pod weight, plant height, leaflet number per pinnate leaf, and pods per plant. Research on the genetic diversity of the two species has revealed a strong correlation (R^2^ = 0.84) between morphological variability and the distinction between *Moringa oleifera* and *Moringa peregrina* [36]. Significant variation (*p* < 0.05) in traits such as plant height and leaf yield has been documented among different accessions, with principal components explaining up to 87% of the total phenotypic variance [37]. Systematic morphological and anatomical analyses have identified traits linked to its physiology; for instance, triangular sclerenchyma arrangements may underpin rapid growth, and the widespread presence of myrosin cells corresponds to its characteristic glucosinolate profile [38]. Several comparative studies on *Moringa* species, including *Moringa oleifera*, *Moringa stenopetala* and *Moringa peregrina*, have identified diagnostic leaf features that can serve as partial phenotypic proxies for genotypic assessment. These features include leaflet shape, trichome density, and petiole morphology [39]. Further divergence was observed in the morphology of inflorescences, pods, and seeds across different genotypes. This provides a phenotypic framework for elucidating the genetic networks that control these traits.

Environmental stress significantly influences the morphological and physiological functions of *Moringa*. Under drought conditions, plants exhibit reduced plant height and stem diameter, limited leaf growth, and significantly increased root length. This may be attributed to root system expansion and proliferation in low-osmotic soil environments to seek and acquire water [40]. Exposure to high temperatures induces a rapid onset of chlorosis and wilting in moringa seedlings, accompanied by extensive cellular damage that progresses to irreversible necrosis. In contrast, exposure to low temperatures provokes only mild leaf curling, while overall tissue integrity remains largely preserved, indicating a considerable degree of cold tolerance in this genus [41]. Despite its notable resilience to saline environments, *Moringa* growth is ultimately inhibited under extreme salt stress. Observed phenotypic manifestations include reductions in leaf size and water uptake capacity and consequent overall declines in plant biomass [42,43].

There has been a growing number of phenomics studies on *Moringa* across different genotypes and environmental conditions; however, these remain largely confined to traditional morphological descriptions and lack the support of modern high-throughput, high-precision technologies. In the future, high-throughput phenotyping platforms based on unmanned aerial vehicles (UAVs) should be utilized, in conjunction with technologies such as multispectral imaging and thermal imaging, to conduct dynamic monitoring of *Moringa*’*s* field growth status, biomass accumulation and stress responses [44]. With the aid of artificial intelligence, genotype–phenotype association models should be constructed to quantify key agronomic traits such as leaf area index and seed yield, thereby providing phenotypic data to support subsequent molecular breeding efforts.

### 3.3. Transcriptomic Analysis Reveals Developmental Regulation

Transcriptomics provides a comprehensive portrayal of gene expression across specific tissues and developmental stages in an organism. Comparative transcriptomic analyses have enabled the identification of differentially expressed genes in distinct *Moringa* species. Although *Moringa oleifera* and *Moringa concanensis* exhibit approximately 80% sequence similarity, they diverge in gene functional enrichment categories [45]. Pasha et al. [46] elucidated the correlation between gene expression and metabolite profiles in various moringa tissues. Candidate genes for quercetin biosynthesis were highly expressed in flowers and leaves, genes associated with mineral metabolism were prominently expressed in leaves, and candidate genes for vacuolar iron and calcium transporters showed higher expression in roots and leaves. These findings provide genomic-level insights into the nutritional traits and water purification functions of *Moringa*.

Regarding growth and development regulation, the *Aux/IAA* family genes participate in the regulatory network of stem regeneration molecules during stem organogenesis in *Moringa* through the auxin signaling pathway [30]. MADS-box genes interact with other genes by encoding transcription factors containing MADS domains, thereby precisely regulating the processes of floral development and seed maturation in *Moringa* [30]. Under different growth conditions, moringa seedlings exhibit a dynamically changing miRNA expression profile; these miRNAs target genes involved in cell division, expansion and hormone signalling pathways, enabling *Moringa* to respond rapidly to environmental changes and optimise resource allocation to maintain a growth advantage [31]. In response to abiotic stress, the endophytic fungal symbionts of *Moringa* enhance the expression of heat-shock factors (HSFs) by upregulating the expression of antioxidant enzymes such as ascorbate peroxidase (APX) [47]. *TPS* genes play a critical role in salt tolerance [48], *MoTPP* and *WRKY* transcription factors have also been identified as genes involved in abiotic stress response [49]. Elucidating the mechanisms of bioactive compounds, MIC-1 suppresses LPS-induced inflammation by modulating multiple signaling pathways associated with inflammatory responses [50]. The antimicrobial peptide MOp2 demonstrates antimicrobial efficacy by regulating bacterial gene expression and disrupting physiological processes [51,52].

Transcriptomic studies on *Moringa*, encompassing diverse tissues and responses to various growth environments, have provided systematic insights into biological processes, but there remains a need to shift toward the study of regulatory networks. Co-expression clusters were constructed using weighted gene co-expression network analysis (WGCNA) to elucidate the transcriptional expression networks of *Moringa* under abiotic stress. These transcriptional regulatory mechanisms provide a molecular basis for the breeding of stress-tolerant germplasm.

### 3.4. Proteomics Reveals Key Pathways

Proteomics provides robust support for elucidating the molecular mechanisms underpinning plant growth, development, metabolite production, and environmental responses. The rich profile of bioactive compounds and diverse bioactivities in *Moringa* underscores the significance of proteomic investigations. The inaugural proteomic analysis of moringa tissues in 2016 identified 202 proteins across four vegetative organs. Heat-shock proteins and flocculation-related proteins (could agglutinate human and rabbit erythrocytes) [53,54] detected in leaves, stems, bark, and roots provided insights into their thermotolerance and potential roles in adsorption and antimicrobial activities [55]. Functional enrichment analysis of proteins identified via 2DLC-MS/MS proteomic profiling of moringa flowers revealed predominant localization in the cytoplasm (72.7%), catalytic activity (61.5%), and macromolecule metabolism (43.7%) [56]. The antimicrobial mechanism of moringa leaf extracts was also elucidated; it induces lysis in *Escherichia coli* by modulating the expression of multiple proteins involved in bacterial biological processes such as stress response, metabolism, and energy homeostasis [57]. The antimicrobial peptide MOp2, isolated from moringa seeds, inhibits *Staphylococcus aureus* by impacting cell wall biosynthesis, leading to oxidative damage, increased membrane permeability, aberrant cell wall formation, reduced protein synthesis, and dysregulated energy metabolism [52,58].

A label-free proteomic study investigating protein alterations during moringa seed germination identified 174 differentially expressed proteins, with a substantial number of catalytic enzymes, including hydrolases, exhibiting high expression during this phase. This finding suggests potential applications in the production of bioactive peptides and in cheese processing [59,60].

Proteomics research on *Moringa* remains relatively limited. In addition to identifying the basic protein composition, particular attention should be paid to the regulatory roles of post-translational modifications, such as phosphorylation and acetylation, on the activity of key enzymes in *Moringa* [61]. Constructing protein interaction networks will help elucidate the flocculation mechanisms of cationic proteins (such as MOCP) in moringa seeds during water purification, as well as the synergistic effects of leaf proteins in the antioxidant defense system, thereby providing a molecular basis for the subsequent development of highly efficient water purifiers and antioxidant functional foods. To provide a consolidated overview, we have summarized the current research on the genes, transcription factors, and protein functions of *Moringa*, as presented in Table 2.

### 3.5. Metabolomic Analysis of Reaction Mechanisms

As the end products of gene expression and protein regulation, metabolites directly reflect an organism’s physiological status. Metabolomics enables the comprehensive identification and quantitative analysis of these small molecules, thereby directly elucidating the mechanisms underlying the formation of biological traits [63]. *Moringa* contains a variety of medicinal and highly nutritious components: it provides all essential and semi-essential amino acids required by the human body [64], as well as vitamins, polyphenols, glucosinolates, and alkaloids [7,64,65,66]. It is a rich source of protein (36%), lipids (38.7%), vitamins (e.g., vitamin E), minerals (e.g., magnesium, copper), and glucosinolates [67]. UPLC analysis targeting key chemical constituents in *Moringa* identified flavonols and phenolic acids as the predominant phenolic compounds in leaves and flowers, with the leaves exhibiting the highest polyphenol concentration [7]. Seeds are also rich in phenolic acids, suggesting that polyphenols may serve as critical or bridging constituents in *Moringa* metabolic processes [68]. The distribution of these bioactive compounds and their associated health benefits varies significantly across plant tissues, as summarized in Table 3.

Significant variations in metabolite composition distinguish different *Moringa* varieties. Flavonoids are recognized for their pronounced pharmacological activities in the genus. Makita et al. [85] employed UPLC-qTOF-MS to analyze three potent flavonoids—rutin, kaempferol glycoside, and isoquercetin glycoside—and revealed that the genetic background partially accounts for quantitative differences in flavonoid content. Comparative nutritional analyses of 13 *Moringa* cultivars by Ndhlala et al. [86] identified variations in carbohydrate, calorie, sugar, dietary fiber, and micronutrient content. Notably, *Moringa oleifera* exhibits lower levels of vitamin E and tocopherol isomers than *Moringa stenopetala*, with substantial divergence in fatty acid profiles [87]. Furthermore, environmental conditions modulate metabolite accumulation. The specific metabolites in Chinese and Indian moringa oils differ, serving as potential biomarkers for cultivar differentiation [88].

The accumulation of specific metabolites reflects adaptive responses and varies markedly depending on the type and intensity of the encountered stress. Under drought conditions, photosynthetic activity in *Moringa* declines, accompanied by an initial increase, followed by a decrease in chlorophyll and carotenoid content [89]. This adaptive mechanism, characterized by the suppression of lipid peroxidation and mitigation of photoinhibition, facilitates the rapid recovery of photosynthetic rates and growth upon rehydration [90]. Fini et al. [91] observed a substantial upregulation in the biosynthesis and emission of volatile organic compounds, such as isoprene, with emission rates tripling as drought severity intensified. This phenomenon is attributed to isoprene production and the accumulation of protective flavonoids, which collectively alleviate the oxidative damage associated with drought stress [90]. Beyond drought, *Moringa* demonstrates robust tolerance to moderate salinity, mediated by enhanced concentrations of antioxidant compounds and the augmented activity of associated enzymes [92]. Extreme temperatures are another critical abiotic regulatory factor. Both high and low temperatures elicit the upregulation of phenolic compounds, including flavonoids and total phenolics, in *Moringa*. However, the functional outcomes of this upregulation differ among different cell types. Under cold conditions, it effectively activates antioxidant defense systems; conversely, under high temperatures, the increased metabolic output is often insufficient to prevent sustained oxidative damage and cell death [41]. Cultivation practices also notably influence the metabolic profile of *Moringa*. Hydroponically grown plants exhibit significantly higher levels of phenolic acids in their leaves than their field-cultivated counterparts [93]. Targeted seed pretreatment has emerged as a promising agronomic strategy. Pretreatment with blue light and ethylene has been shown to precondition the antioxidant and photosynthetic pathways in *Moringa*, thereby enhancing its tolerance to moderate drought [94].

Moringa leaf extract (MLE) is recognized as a plant growth promoter applicable across diverse agricultural systems [95]. Its efficacy in promoting crop development has been demonstrated in various species, including wheat [96], legumes [97], tomato [98], and citrus fruits [99]. Additionally, MLE enhances plant tolerance to abiotic stress. Foliar application of MLE during plant development has been shown to mitigate salt stress in legumes [100]^,^ lettuce [101], and tomato [102]. It also confers enhanced tolerance to extreme temperatures in wheat [103] and moringa itself [104], and alleviates drought stress in mint [105] and tomato [106]. These beneficial effects are attributed to the rich composition of MLE, which includes antioxidants, plant hormones, and essential minerals. These constituents mediate enhanced enzymatic antioxidant activity, thereby improving overall plant growth [107] and stress resilience [95] under adverse environmental conditions.

Metabolomics should focus on enhancing the construction of dynamic metabolic pathways and flux analysis. By utilizing metabolite quantitative trait locus (mQTL) mapping techniques to link variations in metabolite levels to genomic loci, the breeding process can be accelerated. Elucidating the regulatory networks governing the biosynthesis of polyphenols, flavonoids and other compounds in *Moringa* will provide a foundation for the development of new, highly nutritious and high-value *Moringa* varieties.

## 4. Multi-Omics Integration: Advancing the Development of Molecular Breeding in *Moringa*

Plant growth and development are influenced by both genetic information and environmental factors. Multi-omics integration has become an indispensable component of plant research for elucidating complex endogenous biological mechanisms. By integrating genomics, transcriptomics, proteomics, metabolomics, and other omics technologies, researchers can gain profound insights into plant evolution, gene function, and their interactions with the environment [108].

Currently, multi-omics approaches have been systematically applied to numerous species, including humans [109,110], microorganisms [111,112], and microbe–plant symbionts [113]. Multi-omics studies in plants are comparatively more intricate because of complex interaction networks and metabolic diversity [114]. These methods are primarily employed to decipher functional gene mechanisms, thereby enhancing the characterization of gene functions at various regulatory levels [115,116]. Leveraging multi-omics technologies, dedicated omics databases and analytical platforms have been established for many species. The CottonMD database aids in identifying genotype-phenotype associations and candidate gene functions [117], and the Maize Gene Network Map enhances the functional annotation of the maize genome [118]. Similar resources have been developed for Brassica species [119], soybeans [120], and tomatoes [121]. Collectively, these platforms underscore the substantial potential of multi-omics approaches in plant breeding and gene function characterization. By drawing on advances in high-throughput omics technologies applied to other model plants and crops, it is possible to establish a comprehensive framework for future research on *Moringa*, with the aim of elucidating its mechanisms of stress adaptation, the regulatory networks governing the synthesis of secondary metabolites, and the molecular basis of its desirable traits. Integrated multi-omics analyses have been extensively applied in *Moringa* research. The high nutritional value, robust stress tolerance, and rapid growth of *Moringa* are attributed to multilevel regulatory processes involving gene transcription, metabolic transformations, and biosynthetic pathways. A systematic analysis of these pathways facilitates the construction of molecular regulatory networks underpinning these traits. Joint analysis of the microbiome, metabolome, and transcriptome revealed that *Moringa* polysaccharides can reduce inflammatory cytokine expression in patients with ulcerative colitis, indicating their potential as functional foods for preventing ulcerative colitis [122]. Integrated transcriptomics and metabolomics analyses revealed that moringa leaves are more effective than seeds or root bark in ameliorating scopolamine-induced learning and memory deficits in mice [123]. *Moringa* isothiocyanate (MIC-1) effectively reverses some of the epigenetic and transcriptomic abnormalities induced by a high-sugar diet in mice through its antioxidant and anti-inflammatory effects [124].

Regarding nutritional quality, simultaneous evaluation of genotype and metabolomics aids in more refined classification of different *Moringa* genotypes and supports the optimization of germplasm evaluation [36]. Joint analysis of transcriptomics and metabolomics provides a more comprehensive perspective for in-depth understanding of regulatory networks [125]. For the accumulation mechanisms of bioactive compounds in *Moringa*, including vitamins, flavonoids, and glucosinolates, researchers first employ metabolomics to identify metabolites, followed by transcriptomic sequencing and Weighted Gene Co-expression Network Analysis (WGCNA) to establish correlations between metabolite levels and gene expression patterns [126]. This approach facilitates the identification of key transcription factors regulating critical metabolic pathways, with subsequent experiments validating the regulatory roles of these factors. This “gene-to-product accumulation” framework effectively constructs a causal chain for the formation of quality traits, thereby supporting the continuous breeding of superior traits.

**Figure 4:**
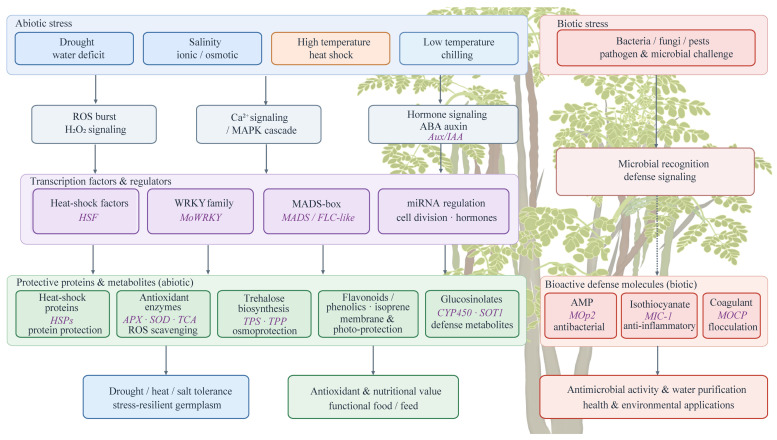
Schematic of the molecular response pathways underlying stress tolerance and bioactivity in *Moringa*. Gene and gene-family names are shown in italics; arrows indicate the direction of information flow.

To provide precise targets for trait improvement, molecular breeding necessitates the integration of the latest advancements in omics fields, including the utilization of phenomics to identify key target traits, metabolomics to characterize biochemical constituents, proteomics to identify proteins catalyzing or regulating the synthesis of these constituents, and transcriptomics to trace upstream regulatory genes. The application of emerging technologies also urgently requires solutions to current challenges. Systems biology can elucidate the complex traits of *Moringa*, but this requires the development of standardized multi-omics databases; WGCNA has currently been used in studies of abiotic stress to identify genes associated with nutritional traits, but the sample size remains small; GWAS can locate genetic loci associated with core complex traits of *Moringa*, but there is a lack of high-density SNP markers. The integration of these technologies holds promise for overcoming these bottlenecks. Moving forward, efforts should focus on strengthening genomic resource development, expanding population sizes, and establishing a multi-omics data-sharing platform. This enables precise and efficient improvement of *Moringa* traits, better meeting current market demands for diverse functions and providing a theoretical basis for breeding adaptable germplasm resources (Figure 4).

## 5. Summary and Outlook

Multi-omics research also provides a theoretical foundation for the subsequent development of *Moringa* across multiple fields. By identifying the accumulation of key metabolites such as flavonoids and phenolic acids, it is possible to develop functional foods targeting antioxidant and anti-inflammatory properties; confirming the pharmacological potential of *Moringa* phytochemicals can accelerate the translation of standardized pharmaceuticals; elucidating the cation-binding proteins in the seeds and their flocculation mechanisms provides a basis for the development of green water purifiers; analysis of how components such as polyphenols and saponins improve livestock growth performance can better support the development of green livestock farming; revealing bioaccumulation characteristics under adverse conditions also provides a theoretical foundation for the breeding of varieties for carbon sequestration and ecological restoration. This comprehensive utilization, from leaves to seeds, has driven the transformation of the *Moringa* industry toward a circular economy model, enhancing its potential for sustainable development. This can only be realized through the effective application of omics-driven approaches during the breeding process.

In the future, the construction of higher-quality pangenomes and the emergence of a comprehensive “pan-omics” framework encompassing phenomics and epigenomics data will bring transformative opportunities. Epigenomics should be utilized to elucidate the core mechanisms by which DNA methylation, histone modifications, and chromatin accessibility regulate gene expression and to reveal the epigenetic networks underlying stress resistance and the synthesis of secondary metabolites [127]; employ single-cell and spatial transcriptomics to analyze tissue heterogeneity and cell fate determination at high resolution, thereby elucidating cell-type-specific regulation of complex traits [128]; conduct pan-genomic studies to systematically identify variants associated with genetic diversity and desirable traits, providing comprehensive resources for molecular breeding [129].

Despite the significant progress in *Moringa* research, several notable limitations persist. Genomic and transcriptomic studies have primarily focused on elucidating stress resistance mechanisms. While key regulatory factors involved in responses to cold, heat, and salinity have been preliminarily identified, the majority of these remain unverified genetically or biochemically. Moreover, research on the regulatory genes and molecular networks governing major metabolite biosynthesis is still largely descriptive or correlative. Historically, the predominant research perspective has viewed *Moringa* as a source of external bioactive additives rather than focusing on the plant’s intrinsic regulatory biology. To comprehensively understand the precise internal regulatory mechanisms of this organism, integrated multi-omics analyses, including metabolomics, proteomics, and other functional omics data, are necessary.

Based on this, future research should prioritize the following:

(1) Taking an application-oriented approach, we will conduct mechanism-oriented research. By leveraging pan-genomic analysis to identify the genes and alleles that regulate key traits in *Moringa*—such as drought and salt tolerance and high nutrient content—we will combine gene editing with molecular marker-assisted selection (MAS) technologies to employ molecular design breeding and develop new *Moringa* varieties adapted to diverse environments. To address diverse industrial needs—including feed, medicinal, and food applications—we will develop specialized varieties and establish standardized cultivation and processing systems, thereby accelerating the transition of *Moringa* from basic research to practical industrial applications. In parallel, because gene-edited and transgenic lines intended for food and feed use are governed by biosafety and regulatory frameworks that differ markedly among countries (for example, between the EU, the USA and China), the development of improved *Moringa* varieties should be accompanied by early consideration of region-specific regulatory, biosafety and traceability requirements [130].
(2) Adopting integrated multi-omics strategies is imperative. While single-omics studies have yielded valuable results, they are insufficient on their own to elucidate the complex and multifaceted functionalities of *Moringa*. To avoid duplication of sequencing and analysis efforts, we should integrate *Moringa* pan-genome resources from diverse global populations, single-cell and spatial transcriptomic cell atlases, epigenomic dynamic regulation maps, and proteomic–metabolomic functional data to establish a unified, open, and standardized multi-omics database that provides shared basic resources and analytical tools for *Moringa* researchers worldwide.

## 6. Conclusions

As a strategic crop for addressing global malnutrition and climate change, *Moringa* breeding is shifting from traditional hybridization methods to multi-omics-driven designed breeding. The integration of a systems biology framework incorporating multiple omics disciplines provides a key technical pathway for elucidating the genetic basis of its complex traits and overcoming breeding bottlenecks. In particular, genomics identifies genetic variation, uncovers rare alleles conferring stress resistance, and locates major effect loci for key agronomic traits; transcriptomics, combined with epigenomics, enables high-resolution analysis of cellular heterogeneity in gene expression, spatial regulation, and the epigenetic mechanisms underlying stress responses; the integration of proteomics and metabolomics further validates functional pathways and provides targets for nutritional improvement. This interdisciplinary research paradigm will comprehensively decode the patterns of *Moringa*’*s* biological processes, providing robust scientific and technological support for the development of new high-yielding, high-quality, and stress-tolerant varieties.

## Figures and Tables

**Table 1:** Comparative analysis of genomic assembly metrics and key features across four versions of the *Moringa oleifera* genome.

Items	Tian (2015)	AOCC v1 (2018)	Shyamli (2021)	AOCC v2 (2022)
Sequencing platform	HiSeq 2500	HiSeq 2000	PacBio Sequel HiSeq 2500	Oxford Nanopore
Genome size	315,160,696 bp (315.16 Mb)	216,759,177 bp (216.76 Mb)	281,946,330 bp (281.95 Mb)	236,366,566 bp (236.37 Mb)
Assembly level	Scaffold	Pseudomolecule	Scaffold	Chromosome
Number of scaffolds	33,332	22,329	915	748
Scaffold N50	1,140,476 bp (1.14 Mb)	957,246 bp (0.96 Mb)	4,719,167 bp (4.72 Mb)	14,962,574 bp (14.96 Mb)
Largest scaffold	6,788,971 bp (6.79 Mb)	4,637,711 bp (4.64 Mb)	13,807,473 bp (13.81 Mb)	30,079,500 bp (30.08 Mb)
GC	NR	36.50%	37.82%	35.70%
Protein coding genes	19,465	18,451	31,056	22,714
Specific gene families	198	172	NR	148
Functional annotated genes	18,299	NR	21,634	16,929

Note: 1,000,000 bp = 1 Mb; all base length values are rounded to two decimal places; “NR” indicates “not reported”.

**Table 2:** Representative functional genes and gene families characterized in *Moringa*, with their associated traits.

Gene/Family	Category	Function/Associated Trait	References
SKP1; F-box genes	Ubiquitination	Lineage-specific families potentially linked to rapid growth	[24]
BAK1	Receptor kinase	Copy-number variation associated with stress adaptation	[27]
HSPs/HSFs	Stress protein	Thermotolerance; drought tolerance via APX up-regulation	[27,47]
MoTPS1 (TPS); TPP	Sugar metabolism	Trehalose-6-phosphate pathway; salt tolerance	[29,48]
CYP450; SOT1	Secondary metabolism	Contracted families; glucosinolate/metabolite biosynthesis	[27]
Aux/IAA	Hormone signaling	Auxin pathway regulating shoot regeneration	[30]
MADS-box (FLC-like)	Transcription factor	Floral development and seed maturation	[62]
MoWRKY	Transcription factor	Differential expression under abiotic stress	[49]
miRNAs	Non-coding RNA	Targeting of cell division, expansion and hormone genes	[31]
MIC-1 (isothiocyanate)	Bioactive metabolite	Anti-inflammatory; modulation of signaling pathways	[50]
MOp2	Antimicrobial peptide	Antibacterial activity against S. aureus	[51,52]
MOCP/lectins	Coagulant protein	Seed flocculation for water purification	[53,54]

**Table 3:** Distribution of bioactive constituents and their associated health benefits across different moringa tissues.

Tissues	Chemical Composition	Functional Properties & Health Benefits	References
leaves	Minerals, vitamins, proteins, amino acids, terpenoids, flavonoids, tannins, saponins, isothiocyanates	Nanoparticle raw materials, water pollution prevention and control, antioxidant, vitamin supplements, immune modulation, constipation relief	[66,69,70,71,72]
Seeds	Lipids, proteins, fats, soluble vitamins, antioxidants, flavonoids, alkaloids, glycosides	water absorption, natural coagulant, preservation, immunomodulatory, anti-inflammatory, insect-resistant	[73,74,75,76,77,78]
Flower	Amino acids, proteins, unsaturated fatty acids	Anti-inflammatory, antioxidant, food additive	[79,80]
Root & stem	Alkaloids, sterols, saponins, phenolics, alkaloids, vitamins	Anti-inflammatory, antifungal, preservation	[81,82,83,84]

## Data Availability

No new data were created or analyzed in this study.
